# Paper-based broadband flexible photodetectors with van der Waals materials

**DOI:** 10.1038/s41598-022-16834-8

**Published:** 2022-07-22

**Authors:** Erfan Mahmoodi, Morteza Hassanpour Amiri, Abdollah Salimi, Riccardo Frisenda, Eduardo Flores, José R. Ares, Isabel J. Ferrer, Andres Castellanos-Gomez, Foad Ghasemi

**Affiliations:** 1 Nanoscale Physics Device Lab (NPDL), Department of Physics, University of Kurdistan, Sanandaj, 66177-15175 Iran; 2 School of Electrical and Computer Engineering, University of Tehran, Tehran, 14395-515 Iran; 3 Department of Chemistry, University of Kurdistan, Sanandaj, 66177-15175 Iran; 4 Research Center for Nanotechnology, University of Kurdistan, Sanandaj, 66177-15175 Iran; 5 Materials Science Factory, Instituto de Ciencia de Materiales de Madrid (ICMM-CSIC), Madrid, 28049 Spain; 6 Centro de Nanociencias y Nanotecnología (CNyN), Universidad Nacional Autónoma de México (UNAM), Ensenada, Baja California 22860 México; 7 Materials of Interest in Renewable Energies Group (MIRE Group), Departamento de Física de Materiales, Universidad Autónoma de Madrid, UAM, Madrid, 28049 Spain; 8 Instituto Nicolás Cabrera, Universidad Autónoma de Madrid, UAM, Madrid, 28049 Spain

**Keywords:** Applied physics, Electronics, photonics and device physics

## Abstract

Layered metal chalcogenide materials are exceptionally appealing in optoelectronic devices thanks to their extraordinary optical properties. Recently, their application as flexible and wearable photodetectors have received a lot of attention. Herein, broadband and high-performance paper-based PDs were established in a very facile and inexpensive method by rubbing molybdenum disulfide and titanium trisulfide crystals on papers. Transferred layers were characterized by SEM, EDX mapping, and Raman analyses, and their optoelectronic properties were evaluated in a wavelength range of 405–810 nm. Although the highest and lowest photoresponsivities were respectively measured for TiS_3_ (1.50 mA/W) and MoS_2_ (1.13 μA/W) PDs, the TiS_3_–MoS_2_ heterostructure not only had a significant photoresponsivity but also showed the highest on/off ratio (1.82) and fast response time (0.96 s) compared with two other PDs. This advantage is due to the band offset formation at the heterojunction, which efficiently separates the photogenerated electron–hole pairs within the heterostructure. Numerical simulation of the introduced PDs also confirmed the superiority of TiS_3_–MoS_2_ heterostructure over the other two PDs and exhibited a good agreement with the experimental results. Finally, MoS_2_ PD demonstrated very high flexibility under applied strain, but TiS_3_ based PDs suffered from its fragility and experience a remarkable drain current reduction at strain larger than ± 0.33%. However, at lower strains, all PDs displayed acceptable performances.

## Introduction

With the development of smart and Internet of Things (IoT) based technologies, great efforts have been made on wearable and flexible electronic devices which are mostly implemented on Polyethylene terephthalate (PET) and Polydimethylsiloxane (PDMS) substrates^1^. However, in addition to high manufacturing costs and lack of biodegradability, their fabrication processes require advanced laboratory equipment, which obstruct their possible practical applications^2^. Flexible paper-based electronic devices are a new class of high-performance devices that offer admirable properties with facile and low-cost fabrication processes, ending in lightweight and environmentally friendly devices that are very promising for the future of smart electronics^3–5^. Flexible photodetectors (PDs), as a member of this family, have received a great deal of attention, suitable for optoelectronic systems such as optical communications, environmental monitoring, and imaging^6^.

Two-dimensional (2D) materials, especially the chalcogenides family, have been increasingly used in optoelectronic devices in recent years due to their adjustable band gap, high electrical conductivity, and effective light-matter interactions^7^. Metal chalcogenide based PDs have shown high photoresponsivity, fast response time, and high quantum efficiency^8^. Interestingly, their paper-based PDs also demonstrates such a high performance^9–11^. Sahatiya et al. fabricated a MoS_2_-Carbon Quantum dot (CQDs) based PD on cellulose paper using a hydrothermal growth of MoS_2_ and casting of CQDs^12^. Their reported device showed a photoresponsivity of 18 mA/W in the visible region with a response time of ~ 0.57 s. Selamneni et al. hydrothermally grown MoS_2_ on cellulose paper followed by decorating with various metal nanoparticles (Au, Pt, and Pd)^11^. The photoresponsivities and response times of the PDs were measured to be around 45–100 mA/W and 0.9–1.2 s in the visible range, respectively. Cordeiro et al. introduced near-infrared PDs by growing MoS_2_ on cellulose paper using a hydrothermal technique. Their PD showed a photoresponsivity of about 200 mA/W and a response time of 3.7 s^13^. However, majority of methods used for the fabrication of paper-based PDs are time-consuming, complex, and consisting wet procedures. In contrast, rubbing of bulk crystal on ordinary paper can be a potential alternative to facile realization of paper-based PDs through a simple, fast and completely dry process. As an example, Mazaheri et al. transferred MoS_2_ flakes by rubbing the corresponding crystal on paper and reported a paper based PD with a photoresponsivity of 1.5 μA/W in a wavelength range of 365 to 940 nm^14^.

In this work, MoS_2_ and TiS_3_ bulk crystals were employed to fabricate paper-based PDs by transferring their corresponding flakes through finger-rubbing process. TiS_3_ is another class of 2D materials with MX_3_ structure, which its unique properties make it very suitable for optoelectronic applications^15^. TiS_3_ is an n-type semiconductor with a direct band gap of 1.1 eV independent of thickness with high reported electron mobility and photoresponsivity^16^. Unlike MX_2_, it has a chain-like structure that provides large aspect ratios with better electrical connectivity even in lower loading fractions^16^. In addition, the electronic band structure of TiS_3_ could be matched with MoS_2_ to provide a suitable heterostructure for optoelectronic applications. Accordingly, three types of MoS_2_, TiS_3_, and TiS_3_–MoS_2_ PDs were fabricated and their optoelectronic properties were carefully evaluated in the wavelength range of 405 to 810 nm. Based on the results, the highest photoresponsivity were measured for TiS_3_ PDs and the lowest for MoS_2_ PDs. In the case of TiS_3_–MoS_2_ (MoS_2_ film was placed on top of the TiS_3_ film and exposed to light), it exhibited a faster response time, and higher on–off ratio thanks to formation of band offset at semiconductor heterojunctions. Numerical simulations also confirmed the superiority of TiS_3_–MoS_2_ PDs over MoS_2_ PDs. Moreover, to evaluate the flexible performance of the PDs, the introduced devices were bent on both positive and negative curvatures and their I_ds_–V_ds_ and photocurrent characteristics were measured under strain range of ± 0.27 to ± 0.54%. The results showed that MoS_2_ PDs had high flexibility, but TiS_3_ based PDs were associated with the decline of drain current and photodetection responsivity at applied strains larger than ± 0.33%.

## Results and discussions

SEM image and XRD analysis of the grown TiS_3_ microcrystals are presented in Fig. S1. Before transferring the flakes, the surface of paper was investigated by SEM and EDX measurements. As can be seen in Fig. S2a, the paper is formed by stacked cellulose fibers and it presents deep gaps/voids between fibers. EDX analysis also confirms the presence of carbon (C), oxygen (O), calcium (Ca), silicon (Si), aluminum (Al), iron (Fe), sodium (Na), and manganese (Mg) in the raw paper where their atomic percentages are listed in the inset of Fig. S2b.

**Figure 1:**
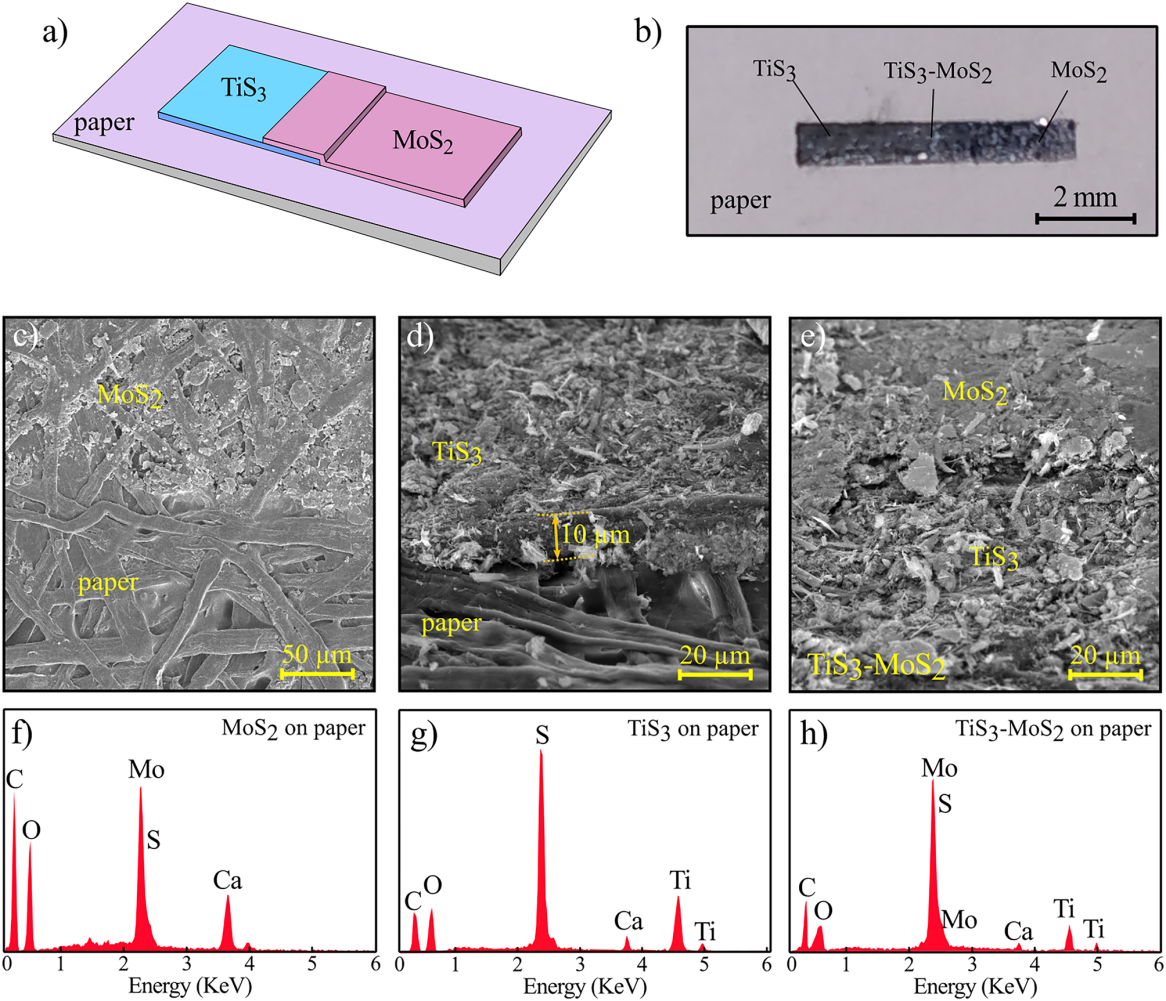
(**a**) Schematic illustration of the TiS_3_-MoS_2_ film on the paper. (**b**) Photograph of the fabricated TiS_3_-MoS_2_ film on the paper. SEM image of the transferred (**c**) MoS_2_, (**d**) TiS_3_, (e) TiS_3_-MoS_2_ flakes on the paper. EDX analysis of the (**f**) MoS_2_, (**g**) TiS_3_, and (**h**) TiS_3_-MoS_2_ films on the paper.

Figure 1 shows the characterization of the deposited flakes on paper substrates. The schematic illustration of the TiS_3_–MoS_2_ heterostructure and its corresponding photograph are also presented in panels (a) and (b) of Fig. 1, respectively. Figure 1c exhibits the SEM image of the MoS_2_ flakes on the paper. According to the figure, MoS_2_ flakes cover the fibers and gaps between fibers of the paper. The deposited TiS_3_ flakes are also presented in Fig. 1d in which the thickness of the film is measured to be ~ 10 μm. Moreover, the SEM image of the TiS_3_–MoS_2_ heterostructure is observed in Fig. 1e. Due to softness and flexibility of MoS_2_, it forms a smoother film than TiS_3_ on paper, which is visible in Fig. 1e. The EDX analysis of MoS_2_, TiS_3_, and TiS_3_–MoS_2_ samples on papers is presented in Fig. 1f,g. By rubbing MoS_2_ on the paper, the Mo and S elements becomes significant (Fig. 1f). The EDX analysis of TiS_3_ sample also proves the presence of S and Ti elements according to Fig. 1g. SEM–EDX analysis of transferred TiS_3_ flakes is also presented in Fig. S3. In the case of TiS_3_–MoS_2_ film, dominant elements are S, Mo, and Ti (Fig. 1h). The C, O and Ca elements come from raw paper. Table S1 summarizes the element contents of all samples with their weight and atomic percentages.

**Figure 2:**
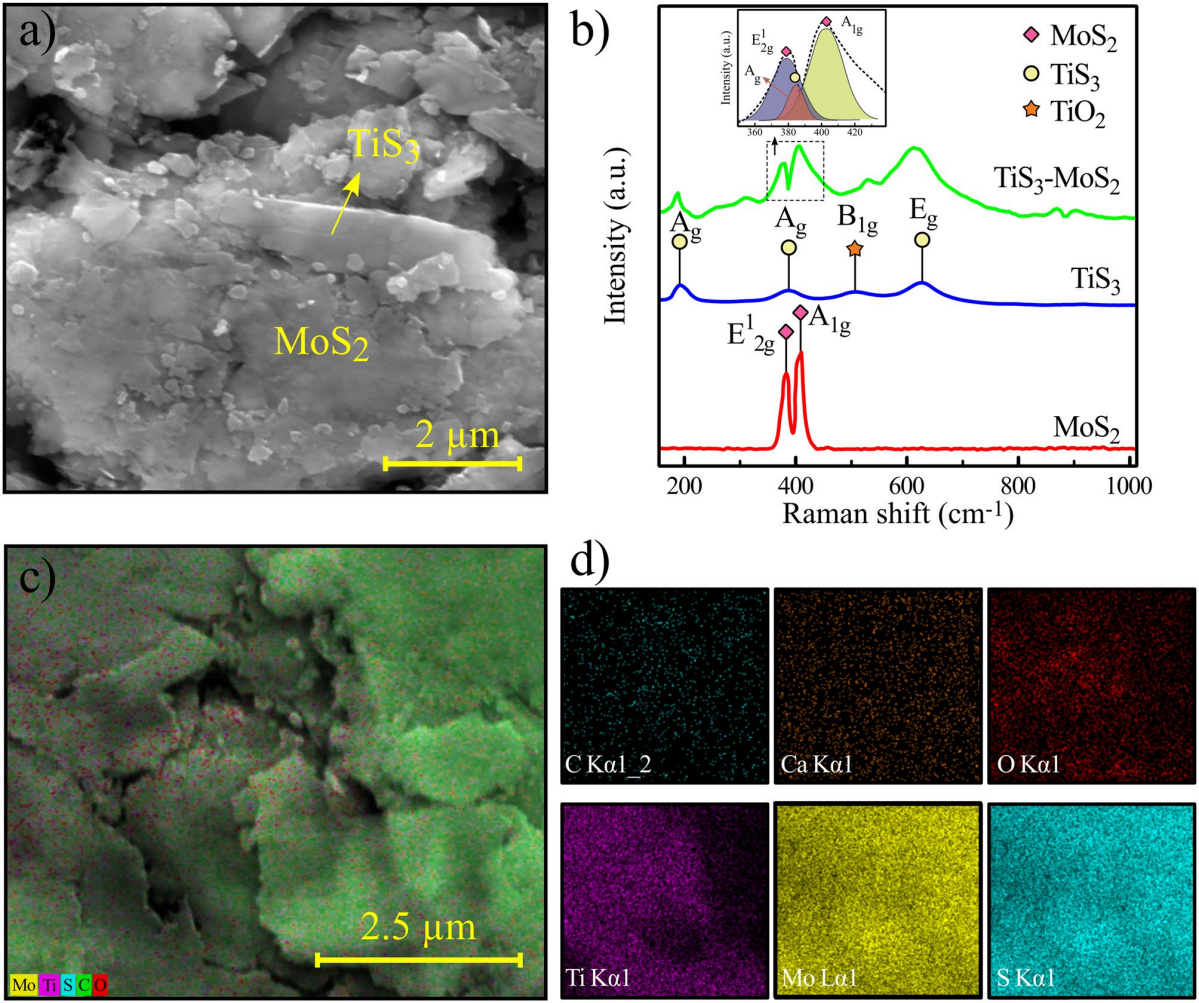
Characterization of the TiS_3_-MoS_2_ film on the paper. (**a**) SEM image of the film. (**b**) Raman spectra of the MoS_2_, TiS_3_, and TiS_3_-MoS_2_ films. (**c**) Mapping analysis of the TiS_3_-MoS_2_ sample on the paper. d) Elemental mapping of six dominant elements in the TiS_3_-MoS_2_ sample.

Figure 2a exhibits the magnified SEM image of the TiS_3_–MoS_2_ film where the MoS_2_ film is deposited on the TiS_3_ film. However, some TiS_3_ microcrystals may also appear on top of the film due to the rubbing process. The Raman spectra of MoS_2_, TiS_3_, and TiS_3_-MoS_2_ samples are also presented in Fig. 2b. For MoS_2_, E^1^_2g_ and A_1g_ peaks are found at 379 and 403 cm^−1^, respectively^17^. In the case of TiS_3_, the 193, 385 and 627 cm^−1^ peaks refer to A_g_ and E_g_ modes, respectively^18^. There is also one additional peak located at 507 cm^−1^, which refers to B_1g_ peak of TiO_2_^19^. This oxide peak may have appeared during the growth of TiS_3_ microcrystals in the ampoule process. The Raman spectrum of TiS_3_–MoS_2_ sample contains all peaks of both structures. The corresponding Ag, E^1^_2g_, and A_1g_ peaks of TiS_3_ and MoS_2_ are also fitted in the Raman spectrum of the hybrid sample. Figure 2c shows the mapping analysis of the TiS_3_–MoS_2_ sample where the dominant elements are separately shown in Fig. 2d. Accordingly, O, C, and Ca elements are due to the raw paper, and elements of Ti, Mo, and S originate from the TiS_3_ and MoS_2_ films. It can be seen that all elements are uniformly distributed on the paper.

**Figure 3:**
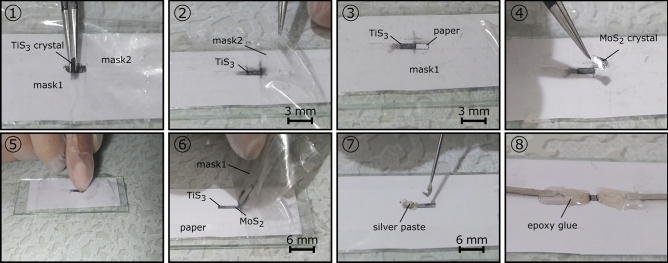
Fabrication steps of the TiS_3_-MoS_2_ PD.

To investigate the optoelectronic properties of the films, MoS_2_, TiS_3_, and TiS_3_-MoS_2_ PDs were fabricated. Details of their fabrication are provided in the experimental section. The fabrication steps of MoS_2_ and TiS_3_ PDs are shown in Figs. S4 and S5, respectively. In the case of TiS_3_–MoS_2_ PD, the fabrication steps are presented in Fig. 3.

**Figure 4:**
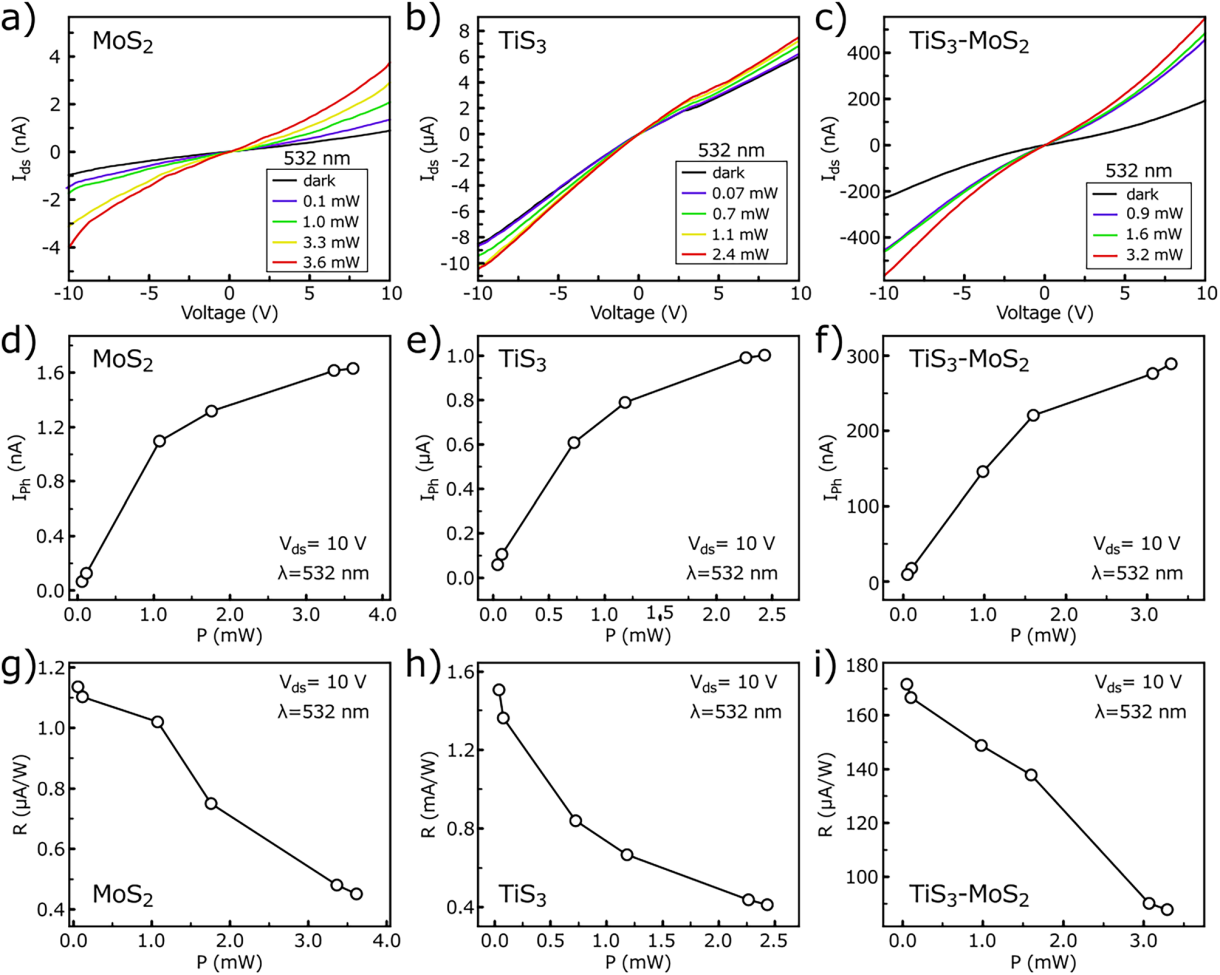
I-V characteristics of the (**a**) MoS_2_, (**b**) TiS_3_, and (**c**) TiS_3_-MoS_2_ PDs under 532 nm laser illumination at different power intensities. Photocurrent versus incident power intensity for (**d**) MoS_2_, (**e**) TiS_3_, and (**f**) TiS_3_-MoS_2_ PDs under 532 nm laser illumination at an applied voltage of 10 V. Photoresponsivity versus incident power intensity for (**g**) MoS_2_, (**h**) TiS_3_, and **i)** TiS_3_-MoS_2_ PDs under 532 nm laser illumination at a supply voltage of 10 V.

Panels (a) to (c) of Fig. 4 display I–V characteristics of the MoS_2_, TiS_3_, and TiS_3_–MoS_2_ PDs at a bias range of − 10 to + 10 V in dark and under 532 nm laser illumination at different power intensities. In all three PDs, significant photocurrents were generated compared to the dark state. The incident power is normalized in terms of laser spot and PD’s channel areas. Accordingly, the effective incident power is calculated as:

1 $${\text{P}}_{{{\text{eff}}}} = {\text{ P}}_{{{\text{laser}}}} \times {\text{A}}_{{{\text{device}}}} /{\text{A}}_{{{\text{laser}}}}$$

where P_laser_ is the power of the laser, A_device_ is the area of the PD’s channel, and A_laser_ is the area of the laser spot. The diameter of the 532 nm laser spot was 2.81 mm and its area (A_laser_) was measured to be 6.22 mm^2^. As the laser intensity increases, the photocurrent also increases for all three PDs. In detail, the drain currents were measured in the range of ~ 0–4 nA, ~ 0–8 μA and ~ 0–450 nA for MoS_2_, TiS_3_, and TiS_3_–MoS_2_ PDs at a biasing voltage of 0–10 V, respectively. Interestingly, the I_ds_ in TiS_3_–MoS_2_ is significantly higher than that of individual MoS_2_, which indicates the effective role of the band offset formation in increasing the photocurrent. The I–V curves of the all PDs in response to 405, 655, and 810 nm laser illuminations are also shown in Fig. S6 where all PDs respond to the laser irradiation by an increase in current. Figure 4d–i compare the photocurrent and photoresponsivity of all three PDs at different power intensities of 532 nm at an applied voltage of 10 V. According to Fig. 4d–f, with increasing effective incident power (from zero to a few mW), increase in the photocurrents are occurred. In the MoS_2_ PD, the photocurrent increases to about 1.6 nA. For TiS_3_, this value is more noticeable and a 1.0 μA increase of photocurrent is observed. In the case of TiS_3_-MoS_2_ PD, photocurrent increases up to 300 nA. The photoresponsivity of the PDs are calculated based on the following equation^20^:

2 $${\text{R}} = {\text{I}}_{{{\text{ph}}}} /{\text{P}}_{{{\text{eff}}}}$$

where I_ph_ is the photocurrent of the PDs. In the MoS_2_ PD, increasing the effective power associates with decrease of the photoresponsivity. The same trends are also observed for TiS_3_, and TiS_3_–MoS_2_ PDs. As the incident power increases, more photocarriers are generated, which increases the recombination rate of photogenerated carriers or the possibility of being captured by the traps, leading to a decrease in R^21,22^. In general, the photoresponsivities are measured in the range of 0.4–1.2 μA/W, 0.4–1.6 mA/W, and 90–170 μA/W for MoS_2_, TiS_3_, and TiS_3_–MoS_2_ PDs, respectively. Accordingly, the photoresponsivity of the last two PDs is three and two orders of magnitude greater than that of MoS_2_ PD.

**Figure 5:**
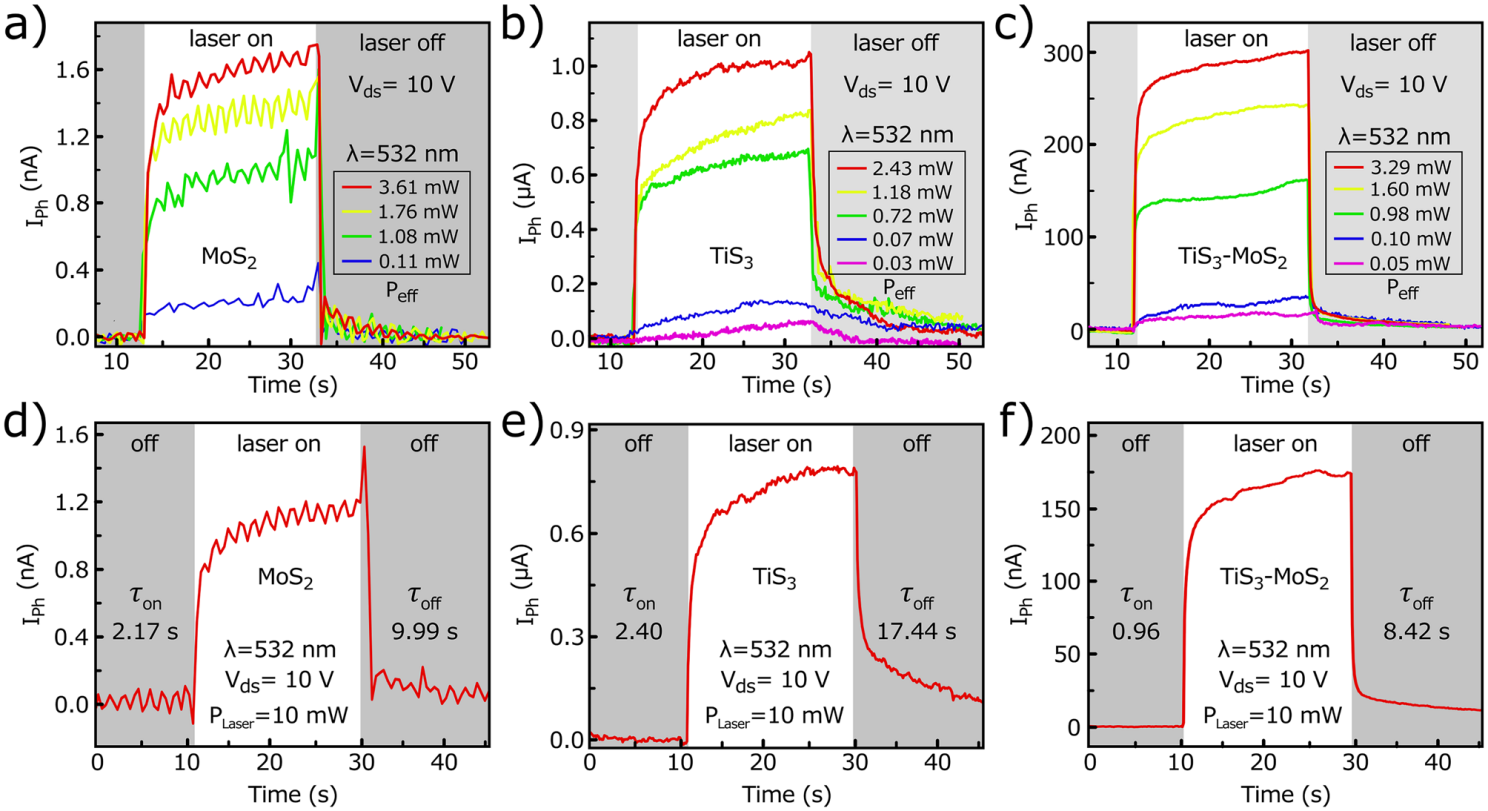
The measured Photocurrent as a function of laser intensities for (**a**) MoS_2_, (**b**) TiS_3_, and (**c**) TiS_3_-MoS_2_ PDs under 532 nm laser illumination at a constant voltage of 10 V. Time trace measurement of (**d**) MoS_2_, (**e**) TiS_3_, and (**f**) TiS_3_-MoS_2_ PDs under 532 nm laser illumination at an applied voltage of 10 V and laser intensity of 10 mW. The corresponding P_eff_ was calculated to be 1.53 mW, 1.03 mW, and 1.39 mW for panels (d) to (f), respectively.

Figure 5a–c show the measured photocurrent in terms of the different effective powers of the 532 nm laser. Accordingly, an increase in the optical powers leads to an increase in photocurrent in all PDs. Panels (d) to (f) of Fig. 5 display the time trace response of the PDs to 532 nm laser illumination at a drain voltage of 10 V at an incident power of 10 mW. Rise time (τ_on_) is defined as the time taken to increase the current from 10 to 90% of its baseline under laser illumination^23^. The fall time (τ_off_) is the time required to reduce the current from 90 to 10% of its baseline^24^. For MoS_2_, the rise and fall times are measured to be 2.17 and 9.99 s, respectively. In the TiS_3_ PD, the τ_on_ and τ_off_ of 2.4 and 17.44 s are measured and in the TiS_3_–MoS_2_ PD, τ_on_ and τ_off_ of about 0.96 and 8.42 s are calculated that it shows faster switching performance compared with two other PDs. In the hybrid PD, MoS_2_ is at the top and is more exposed to light, so the final behavior of the hybrid is more tended toward MoS_2_ than TiS_3_. For this reason, its photoresponsivity is associated with improvement compared with the MoS_2_ PD due to the formation of band offset and efficient charge separation. Moreover, the channel length of TiS_3_ is about 1 mm in the hybrid PD, which is half that of individual TiS_3_ PD. Hence, the needle-like TiS_3_ flake has lower series resistance and therefore shows a faster response time^25,26^. Furthermore, due to the growth process, TiS_3_ probably has more defects than MoS_2_, so it has a slower response time^27^. However, some part of the TiS_3_ is buried under (passivated by) MoS_2_ in the hybrid PD and is resulted in less ambient gas absorption, which speeds up the response time^27^.

**Figure 6:**
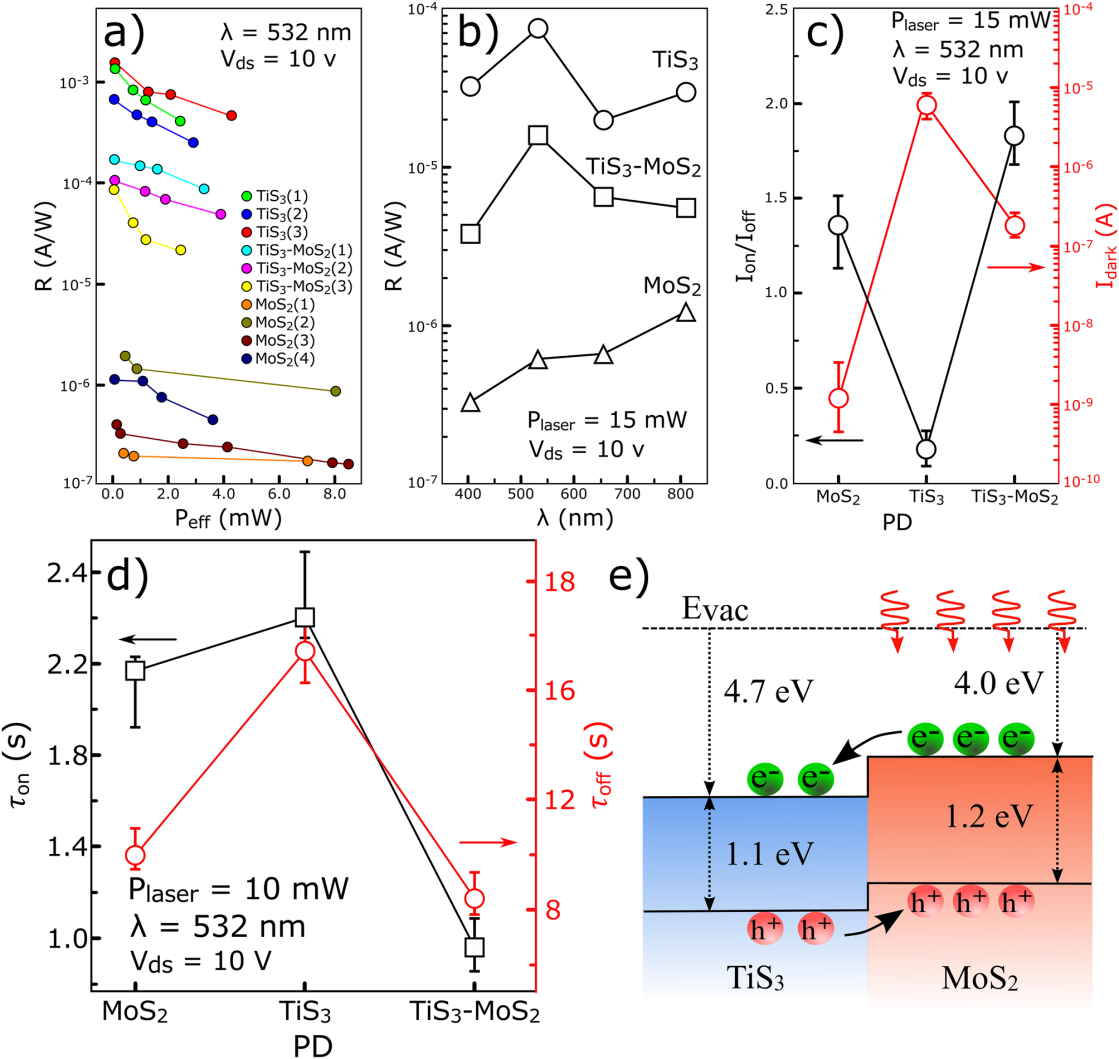
Comparison of (**a**) photoresponsivity as a function of the incident power for several fabricated PDs at an applied voltage of 10 V under a laser wavelength of 532 nm, (**b**) the photoresponsivity of fabricated PDs under various laser wavelengths at an applied voltage of 10 V, (**c**) I_on_/I_off_, and dark current, (**d**) rise and fall times of the MoS_2_, TiS_3_, TiS_3_-MoS_2_ PDs at a constant voltage of 10 V, and (**e**) Energy band diagram and detection mechanism in the TiS_3_-MoS_2_ heterojunction.

Figure 6a compares the photoresponsivity of several fabricated PDs at an applied voltage of 10 V under a laser wavelength of 532 nm. It is observed that all three TiS_3_ PDs have the highest photoresponsivity, followed by TiS_3_–MoS_2_ and MoS_2_ samples, respectively. In general, the R are measured in the range of 0.67–1.56 mA/W for TiS_3_ PDs and 0.20–1.93 μA/W in the case of MoS_2_ PDs. For TiS_3_–MoS_2_ PDs, these values are measured in the range of 0.08 to 0.19 mA/W. Accordingly, MoS_2_ has the lowest photoresponsivity and TiS_3_ possess the highest photoresponsivity. However, both photoresponsivity and photocurrent of TiS_3_–MoS_2_ PDs are considerable compared to the MoS_2_ PD. Moreover, a similar trend is observed in the order of magnitude of all calculated R in these PDs. Figure 6b shows the photoresponsivity of all three devices in terms of different laser wavelengths at the same power of 15 mW and supply voltage of 10 V. All three PDs have a broad response within the range of 400 to 800 nm. The photoresponsivity of the TiS_3_ PDs are one order of magnitude greater than that of the TiS_3_–MoS_2_ PDs and two orders greater than that of the MoS_2_ PDs. Figure 6c compares the measured I_on_/I_off_ ratio and dark current of all three PDs under 532 nm laser irradiation. The error line shows the deviation of the values obtained for the same PDs. The on–off ratio (I_on_/I_off_) is usually used to describe the signal-to-noise ratio^28^. According to Fig. 6c, the values of 1.35, 0.17, and 1.82 are obtained for MoS_2_, TiS_3_, and TiS_3_–MoS_2_ PDs, respectively. Based on them, the highest signal-to-noise ratio is related to the TiS_3_–MoS_2_ PDs. Interestingly, the dark currents are lowest in MoS_2_ (~ 1.2 nA) and highest in TiS_3_ (~ 6.0 μA). Since the light detection mechanism is based on the change of drain current in these PDs, less dark current provides better performance in light detection as will be discussed below^29^. Another important parameters are the rise and fall times which are presented in Fig. 6d. Accordingly, the TiS_3_-MoS_2_ PD has the fastest response time to laser radiation than the other two PDs. Figure 6e shows the energy band diagram of the TiS_3_–MoS_2_ heterostructure and its corresponding photodetection mechanism. TiS_3_ is an n-type semiconductor with an energy band gap of 1.1 eV and MoS_2_ is also an n-type semiconductor with an energy band gap in the range of 1.2–1.8 eV^30,31^. Here, the energy band gap of bulk MoS_2_ is considered because most layers are thick, although the deposited film can contain single and few layers of MoS_2_. The electron affinities of MoS_2_ and TiS_3_ are around 4 and 4.7 eV, respectively, and hence the conduction band of TiS_3_ is located of MoS_2_, forming an n–n^+^ heterostructure^32,33^. In the MoS_2_ film under laser irradiation, electron–hole pairs are generated and the photogenerated electrons enter into the TiS_3_ due to its lower conduction band which prevents electrons from recombination with the holes. Moreover, the valence band of MoS_2_ is located higher than that of TiS_3_ resulting in transferring of minority holes from TiS_3_ into MoS_2_. Hence, the recombination rate of the carriers is decreased in the heterostructure. Such charge separation in the heterostructure is responsible for faster response time, and improved photoresponsivity of the heterostructure compared with individual MoS_2_ PDs.

In order to investigate the effect of thicknesses, the performance of the three hybrid PDs was evaluated with three different TiS_3_ thicknesses. In this regard, thicknesses of ~ 2, ~ 10 and ~ 20 µm were prepared where dark current and photoresponsivity of the PDs were measured as reported in the Fig. S7. Based on the result, as the thickness of TiS_3_ increases, the dark current increases but the photoresponsivity decreases. Less thickness of TiS_3_ leads to more changes in electrical current, which can be due to non-uniformity of the deposited film. Moreover, as the thickness increases, the upper layers are more involved in light absorption and the lower layers play a lesser role in the photogenerated carriers. Therefore, the ratio of photogenerated to non-photogenerated carriers decreases, which leads to a decrease in photoresponsivity. As can be seen, the thickness of ~ 10 µm generally shows better performance compared to less and more thicknesses.

To evaluate the air stability of the PDs, the performance of the fresh devices was also investigated after two weeks based on the measured photocurrent. Based on the Fig. S8, it can be seen that after two weeks of device life, the performance of the PDs is very close to the fresh states, which show their high environmental stability.

Since the substrate is made of paper, the performance of the introduced PDs was thoroughly investigated under applied strain. For this purpose, a homemade setup was fabricated to apply strain to the substrates as shown in Fig. S9. This setup includes a cage covered with aluminum foil to act as a Faraday cage. The sample is fixed to a motor shaft through a hook to provide upward and downward strains by moving in a clockwise or counter-clockwise direction. The strain applied to the PDs was calculated according to ε = t/2R equation, where t is the thickness of the substrate and r is the radius of bending curvature^34^. Figure S10 presents the applied strains in the PDs, which are performed in the range of − 0.54% to + 0.54%˚. As can be seen, at strains larger than ± 0.33%, a significant bending is occurred in the substrates.

**Figure 7:**
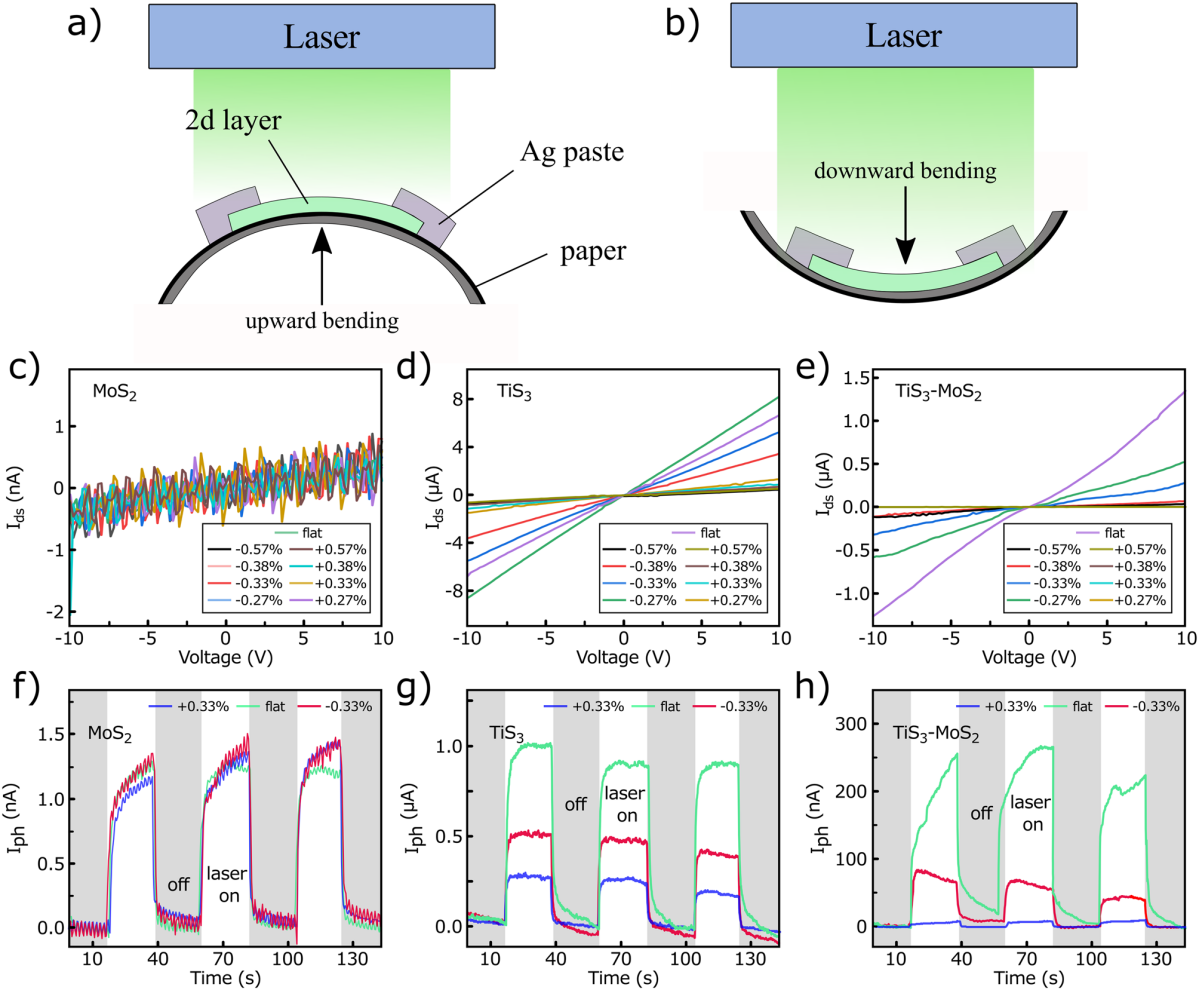
Schematic illustrations of (**a**) upward and (**b**) downward applied strains at the PDs under laser illuminations. I_ds_-V curves of the (**c**) MoS_2_, (**d**) TiS_3_, and (**e**) TiS_3_-MoS_2_ PDs under different applied strains. Photocurrent switching performance of the (**f**) MoS_2_, (**g**) TiS_3_, and (**h**) TiS_3_-MoS_2_ PDs in flat state and at applied strains of ± 0.33% under 532 nm laser illumination at a bias voltage of 10 V.

Figure 7a and b show the schematic of applying the upward (uniaxial tensile) and downward (compression) strains in the PDs under exposing to 532 nm laser illuminations, respectively. Panels (c), (d), and (e) of Fig. 7 compare the I_ds_-V curves of the MoS_2_, TiS_3_, and TiS_3_–MoS_2_ PDs in the flat state and under negative and positive strains, respectively. For the MoS_2_ sample, it is observed that the drain current in the flat state almost remains the same as under different applied strains without any significant change. The flexible and soft structure of MoS_2_ is the main reason for this behavior^35^. Moreover, due to the polycrystalline structure of MoS_2_ flakes and their different thicknesses, the applied strain has a negligible effect on the channel current^36^. In the case of TiS_3_, it is found that the I_ds_ decreases after applying downward strains, but a more significant decrease is observed in the upward strains. Moreover, after upward strain, by returning the TiS_3_ to its flat position, current does not return to its original value of before any strain. The structure of the needle-like TiS_3_ flake is more fragile than that of MoS_2_, and bending can break the contact between TiS_3_ and Ag paste, affecting the conductivity^37^. As a result, even after the sample has returned to the flat state, the current dramatically suffers from decline. In detail, by applying + 0.57% strain, a relative change of drain current |ΔI/I_flat_| is measured to be ~ 88% compared to the flat state, which indicates the high fragility of the TiS_3_ flakes within the channel. A similar trend is observed in the TiS_3_–MoS_2_ PD, but with a more noticeable decrease after positive strains, which could be due to the separation of the two films at the interface and the reduction of the electric field in the channel.

Figure 7f–h show the photocurrent characteristics of all PDs under 532 nm laser irradiation at a drain voltage of 10 V and an incident power of 15 mW. For MoS_2_ PD, the photocurrent is well switched under negative and positive strains of 0.33% similar to the flat state. The lack of change in the photocurrent as well as the slight change in the drain current under the applied strain indicate that the tensile and compressive strains have no effect on the band gap, density of state of carriers, and barrier height at the contacts of the MoS_2_ PDs. This is probably due to the presence of MoS_2_ flakes of different thicknesses and their polycrystalline nature which minimize the effect of strain^36^. In the case of TiS_3_, the photocurrent is about 1.00 μA in the flat state, which is reduced to 0.50 and 0.25 μA in the applied strains of − 0.33 and + 0.33%. This reduction in photocurrent is not due to a change in the energy gap or piezoresistive effect, but rather to the breaking of the needle-like TiS_3_ flakes in the channel, which leads to their less contribution in the drain current and generation of photocarriers. For TiS_3_-MoS_2_, the current is about 250 nA in the flat state, which is reduced to ~ 70 and ~ 10 nA by applying positive and negative strains. As discussed, the brittle structure of TiS_3_ as well as the separation of the two films at the junction, especially at a strain of + 0.33% are the main factors in the TiS_3_–MoS_2_ PD performance drop under applied strains.

**Figure 8:**
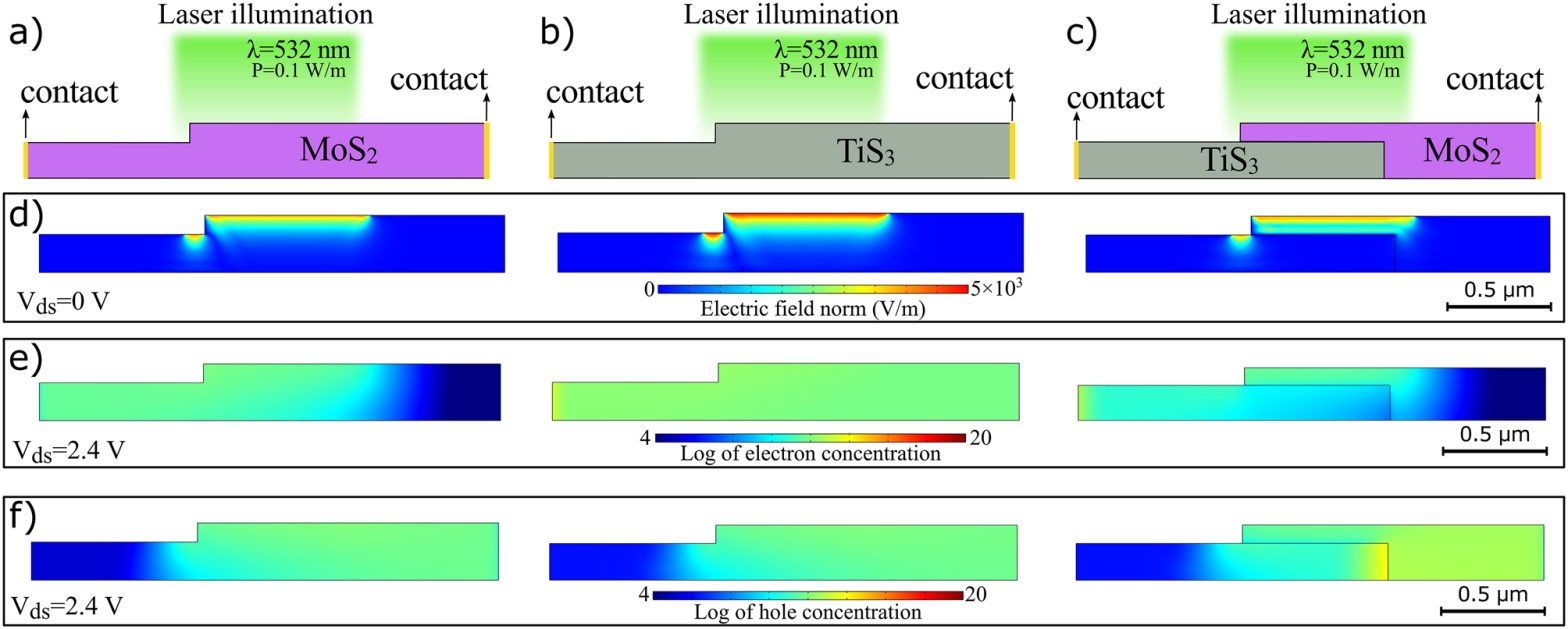
Schematic illustrations of device geometries for (**a**) MoS_2_ (**b**) TiS_3_ and (**c**) TiS_3_-MoS_2_ heterostructure, (**d**) exposed surface of the devices and light-induced electric field, (**e**) electron and (**f**) hole concentrations in a logarithmic scale for the three devices.

Numerical simulation is employed to present further insight about the physics of light-induced generation-recombination processes for charged carriers in these devices. We used the model to calculate the photocurrent of the devices in response to the incident light under various biasing conditions. Details of the simulation can be found in the supporting information section. Panels (a) to (c) of Fig. 8 depict the schematic of the devices consisting MoS_2_, TiS_3_ and heterostructure with stacked layers of both materials. Overlapped part in the heterostructure device was exposed to the incident light with the wavelength of 532 nm. Stray electromagnetic field due to unintentional exposure is also took into the account. Corresponding light-induced electric field can be seen in Fig. 8d for all devices due to laser exposure. Vertical electric field was established by applying a voltage difference between the two terminals at both ends, where contacts are located. Figure 8e–f show the electron and hole concentrations under this biasing condition in a logarithmic scale, respectively. As can be seen, the distribution of electrons and holes in the two structures of MoS_2_ and TiS_3_ is almost uniform, but in the TiS_3_–MoS_2_ device, the band offset accelerates the separation of electrons and holes and causes a non-uniform distribution of carriers at the two-layer boundary. These results indicate that the formed n–n + heterostructure can more efficiently separate the charge and confirms its superior optoelectronic performance compared with the individual MoS_2_ case.

At various light intensities, I–V characteristic curves has been simulated for MoS_2_, TiS_3_, and TiS_3_–MoS_2_ photodetectors. The obtained curves for drain currents are consistent with the measurements as illustrated in Fig. S11a–c. Relative differences between the amplitude of photocurrent is in all devices is in total agreement with the experiments where TiS_3_ and MoS_2_ PDs have higher and lower photocurrents, respect to the hybrid structure under identical biasing and light exposure conditions.

Table 1 compares the performance of the introduced PDs with some other reported paper-based PDs. According to it, most of them have a photoresponsivity in the range of a few μA/W. In the case of MoS_2_, it is observed that our introduced PD has a slightly higher photoresponsivity than the others. The TiS_3_ PD shows a much higher photoresponsivity (in the range of mA/W), which has a significant improvement in performance compared to other reported PDs. However, it shows a larger dark current and a smaller on/off ratio than MoS_2_ PDs which can limit its optical detection performance. Finally, the photoresponsivity of the TiS_3_–MoS_2_ heterostructure shows a significant improvement, which indicates its superiority over other works.

**Table 1:** Comparison of the performance of introduced paper-based PDs with other PDs based on different 2D materials.

Paper-based PDs	V_ds_ (V)	λ (nm)	R (μA/W)	Ref
Te-TiO_2_	0	400	30	^38^
ZnS-MoS_2_	1	554	18	^39^
MoTe_2_	1	532	1.1	^40^
MoSe_2_	1	532	0.2	
WS_2_	1	532	0.06	
MoS_2_	1	532	0.02	
MoS_2_	20	532	1.1	^14^
MoS_2_	10	532	1.2	This work
TiS_3_	10	532	1600	
TiS_3_-MoS_2_	10	532	170	

### Conclusions

The two-dimensional (2D) layered family of transition metals chalcogenides shows high potential as photodetectors because they not only interact effectively with light but also provide high carrier transport properties. In this report, a very simple, fast and promising clean method is introduced to fabricate the paper-based photodetectors using MoS_2_, TiS_3_ and their integration. The all introduced photodetectors show remarkable photoresponsivities in the range of 405 to 810 nm. In the case of TiS_3_–MoS_2_ heterostructure, it associates with fast response time and large on–off ratio compared to individual MoS_2_ and TiS_3_ photodetectors. The numerical simulation results are consistent with the experimental results and confirm the superiority of the hybrid structure over the two other PDs. Moreover, the bending results of the photodetectors indicate that in applied strains smaller than ± 0.33%, these devices still show acceptable performance.

## Methods

### Material

Naturally occurring molybdenite crystal, from Wolfram Camp Mine, Queensland, Australia, were used. Silver paste was purchased from Dycotec Materials, UK DM-SIP-3060S. Ordinary papers (CopiMax, Thailand) were used as substrates.

### Growth of TiS_3_ microcrystals

TiS_3_ microcrystals were synthesized by a solid–gas reaction of titanium powder through direct sulfurization. In detail, titanium powder (Goodfellow, > 99% purity) and sulfur powder (Merck, > 99.9% purity) were vacuum sealed in an ampule and heated up to 500 °C for 20 h. Then, the ampule was cooled down to room temperature and grown microcrystals were collected.

### Fabrication of MoS_2_ (TiS_3_) paper-based photodetector

First, a piece of paper with 2 × 6 cm^2^ dimensions was cut and attached on a slide glass using adhesive tape. A shadow mask (a 1 × 6 mm^2^ rectangular window) was then opened in the center of the paper with adhesive tape. A piece of bulk MoS_2_ (or TiS_3_) was rubbed several times along the length and width of the open-window reign. After deposition of a homogeneous layer of MoS_2_ (TiS_3_), the mask was removed from the paper. Silver paste was then placed on the both sides of the deposited film, and two pieces of soft copper wire were mounted on the paste and kept at room temperature for 24 h for improving the adhesion. Then, the dried silver paste was covered with epoxy glue for strengthening and achieving higher mechanical stability. The photograph of the fabrication steps of MoS_2_ and TiS_3_ PDs are presented in the supporting file.

### Fabrication of TiS_3_-MoS_2_ paper-based photodetector

First, a piece of paper with a dimension of 2 × 6 cm^2^ was cut and attached to a slide glass. A rectangular shadow mask (mask 1) was opened by the adhesive tape in the center of the paper. With the use of another mask (mask 2), this part was divided into two sections with dimensions of 1 × 4 mm^2^ (for TiS_3_ deposition) and 1 × 2 mm^2^ (for MoS_2_ deposition). A piece of bulk TiS_3_ was rubbed several times in the open-window region. Then, mask 2 was removed from the paper and MoS_2_ crystal was finger-rubbed several times on the 1 × 2 mm^2^ part of the bare paper and 1 × 1 mm^2^ part of the deposited TiS_3_. Thus, the center of the sample (device) with dimensions of 1 × 1 mm^2^ was a film composed of TiS_3_ and MoS_2_, in which TiS_3_ was placed beneath the MoS_2_ film. After removing mask 1 from the paper, a homogeneous film composed of TiS_3_, MoS_2_ and, TiS_3_–MoS_2_ with dimensions of 1 × 3, 1 × 1, and 1 × 1 mm^2^ can be achieved, respectively. Similar to previous PDs, copper wires were added to both sides of the film. The TiS_3_-MoS_2_ PD is ready for testing.

### Characterizations

SEM images and EDX analyses were taken by TESCAN MIRA 3 and EDX (Energy-dispersive X-ray) systems. Crystalline structure was identified by using a Panalytical X`Pert Pro X-ray diffractometer at glancing angle configuration (incident angle of 1.7°) with CuKα radiation. Raman spectra of the transferred flakes were measured by Avantes (AVASPEC-ULS3648-RS) system under 532 nm laser illumination. Electrical measurements were carried out by a Keithley 2450 source meter. Optical measurements were done under different laser excitations including 405, 532, 655, and 810 nm at room temperature in air ambient. Bending tests were performed with a homemade setup capable of applying both upward and strains.

## Supplementary Information

Supplementary Information.

## Data Availability

The datasets generated and/or analysed during the current study are not publicly available due to privacy or ethical restrictions. But are available from the corresponding author on reasonable request.
