# Supplementary material for: Paper-based broadband flexible photodetectors with van der Waals materials
Source: Sci Rep. 2022 Jul 22;12:12585. doi: 10.1038/s41598-022-16834-8 (PMC9307754; doi:10.1038/s41598-022-16834-8)
Supplement: Supplementary file 1 — Supplementary Information. [file 41598_2022_16834_MOESM1_ESM.docx]

**Paper-based Broadband Flexible Photodetector with van der Waals materials**

Erfan Mahmoodi^1^, Morteza Hassanpour Amiri^2^, Abdollah Salimi^3.4^, Riccardo Frisenda^5^, Eduardo Flores^6^ , José R. Ares^7^, [Isabel J. Ferrer](https://sciprofiles.com/profile/744588)^7,8^, Andres Castellanos-Gomez^5^_,_ Foad Ghasemi^1*^

^1^Nanoscale Physics Device Lab (NPDL), Department of Physics, University of Kurdistan, Sanandaj, Iran, 66177-15175.

^2^School of Electrical and Computer Engineering, University of Tehran, Tehran, Iran, 14395-515.

^3^Department of Chemistry, University of Kurdistan, Sanandaj, Iran 66177-15175.

^4^Research Center for Nanotechnology, University of Kurdistan, Sanandaj, Iran 66177-15175.

^5^Materials Science Factory, Instituto de Ciencia de Materiales de Madrid (ICMM-CSIC), E-28049 Madrid, Spain.

^6^Centro de Nanociencias y Nanotecnología (CNyN), Universidad Nacional Autónoma de México (UNAM), Ensenada, Baja California C.P. 22860, México.

^7^Materials of Interest in Renewable Energies Group (MIRE Group), Dpto. de Física de Materiales, Universidad Autónoma de Madrid, UAM, E-28049 Madrid, Spain.

^8^Instituto Nicolás Cabrera, Universidad Autónoma de Madrid, UAM, E-28049 Madrid, Spain.

*Corresponding author: [F.Ghasemi@uok.ac.ir](mailto:F.Ghasemi@uok.ac.ir)


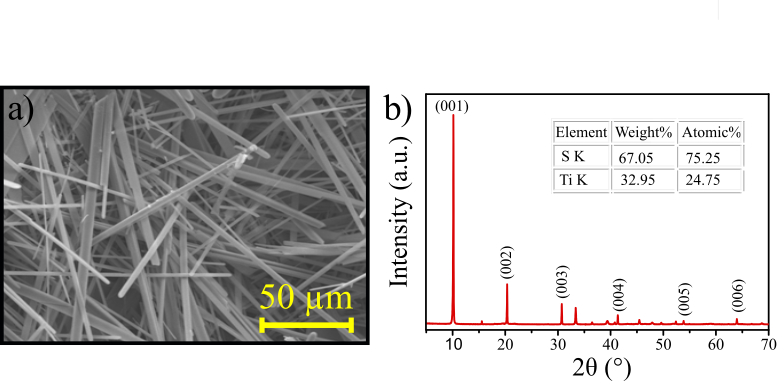


**Figure S1.** **a)** SEM image of the grown TiS_3_ microcrystals. **b)** XRD analysis of the TiS_3_ microcrystals. **Inset:** Corresponding element percentages obtained from EDX analysis.


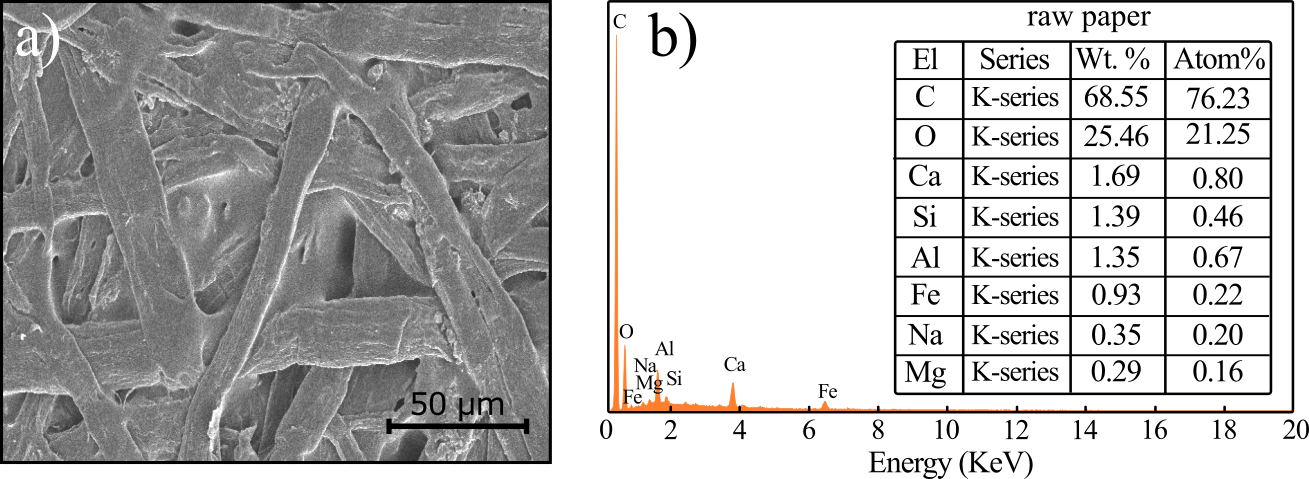


**Figure S2. a)** SEM image of a bare paper surface with several micro-cracks. **b)** EDX spectrum and corresponding element contents in the raw paper.


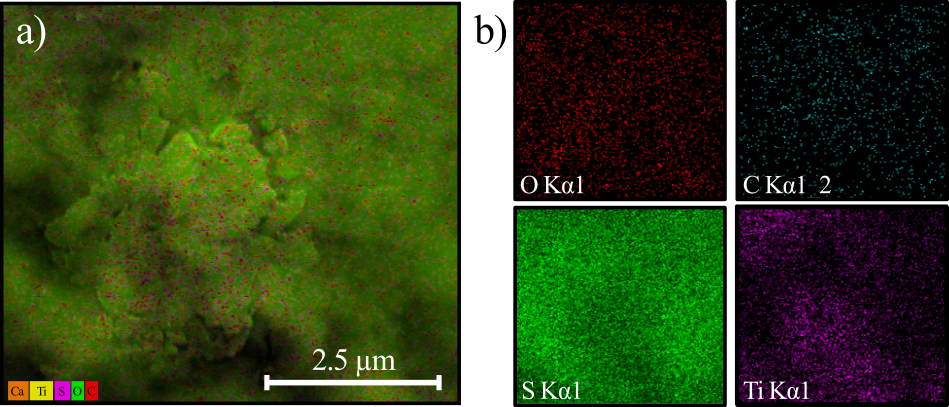


**Figure S3. a)** SEM**-**EDX mapping analysis of TiS_3_ film on paper and **b)** corresponding mapping analysis of its dominant elements.

**Table S1.** Weight and atomic percentages of elements in the MoS_2_, TiS_3_, and TiS_3_-MoS_2_ samples.

|  |  | MoS_2_ | | TiS_3_ | | TiS_3_-MoS_2_ | |
| --- | --- | --- | --- | --- | --- | --- | --- |
| Element | Line Type | Wt% | Atomic % | Wt% | Atomic % | Wt% | Atomic % |
| C | K series | 34.56 | 35.01 | 27.23 | 45.01 | 15.82 | 39.05 |
| O | K series | 34.01 | 18.78 | 23.19 | 28.78 | 5.61 | 10.41 |
| Ca | K series | 3.84 | 0.68 | 1.37 | 0.68 | 0.29 | 0.22 |
| S | K series | 15.81 | 23.73 | 27.14 | 16.81 | 37.37 | 34.57 |
| Mo | L series | 11.78 | 21.80 | 0.00 | 0.00 | 30.88 | 9.55 |
| Ti | K series | 0.00 | 0.00 | 21.06 | 8.73 | 10.02 | 6.21 |
| Total | | 100.00 | 100.00 | 100.00 | 100.00 | 100.00 | 100.00 |


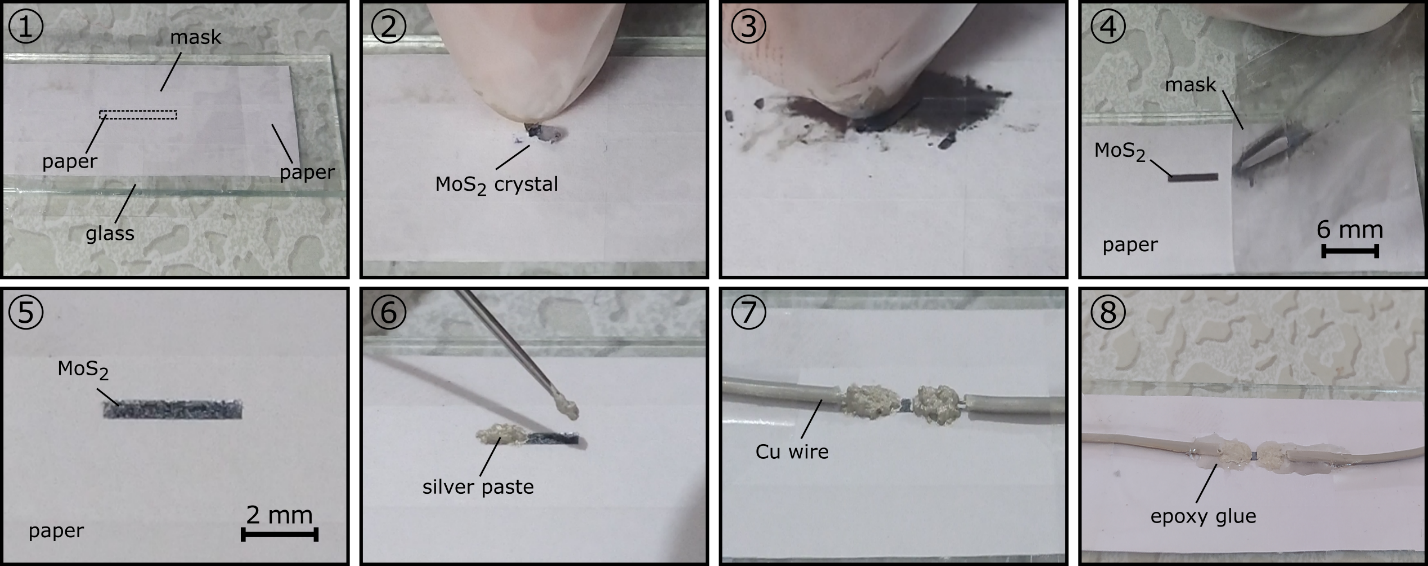


**Figure S4.** Fabrication steps of the MoS_2_ PD.


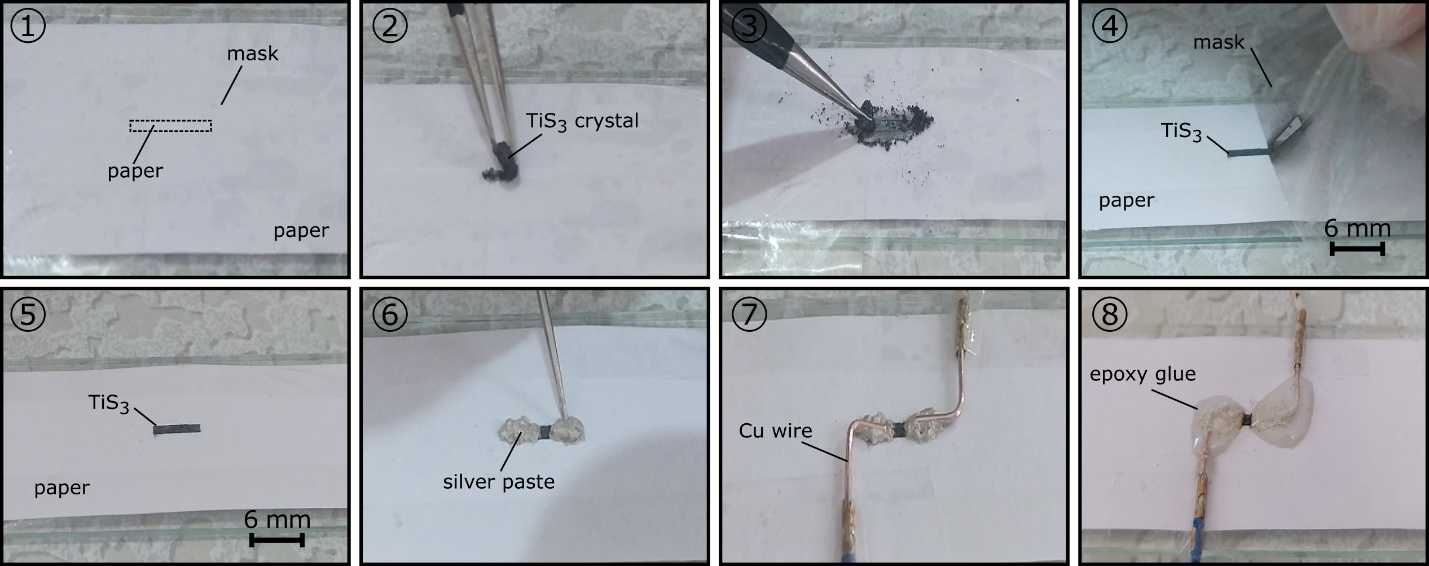


**Figure S5.** Fabrication steps of the TiS_3_ PD.


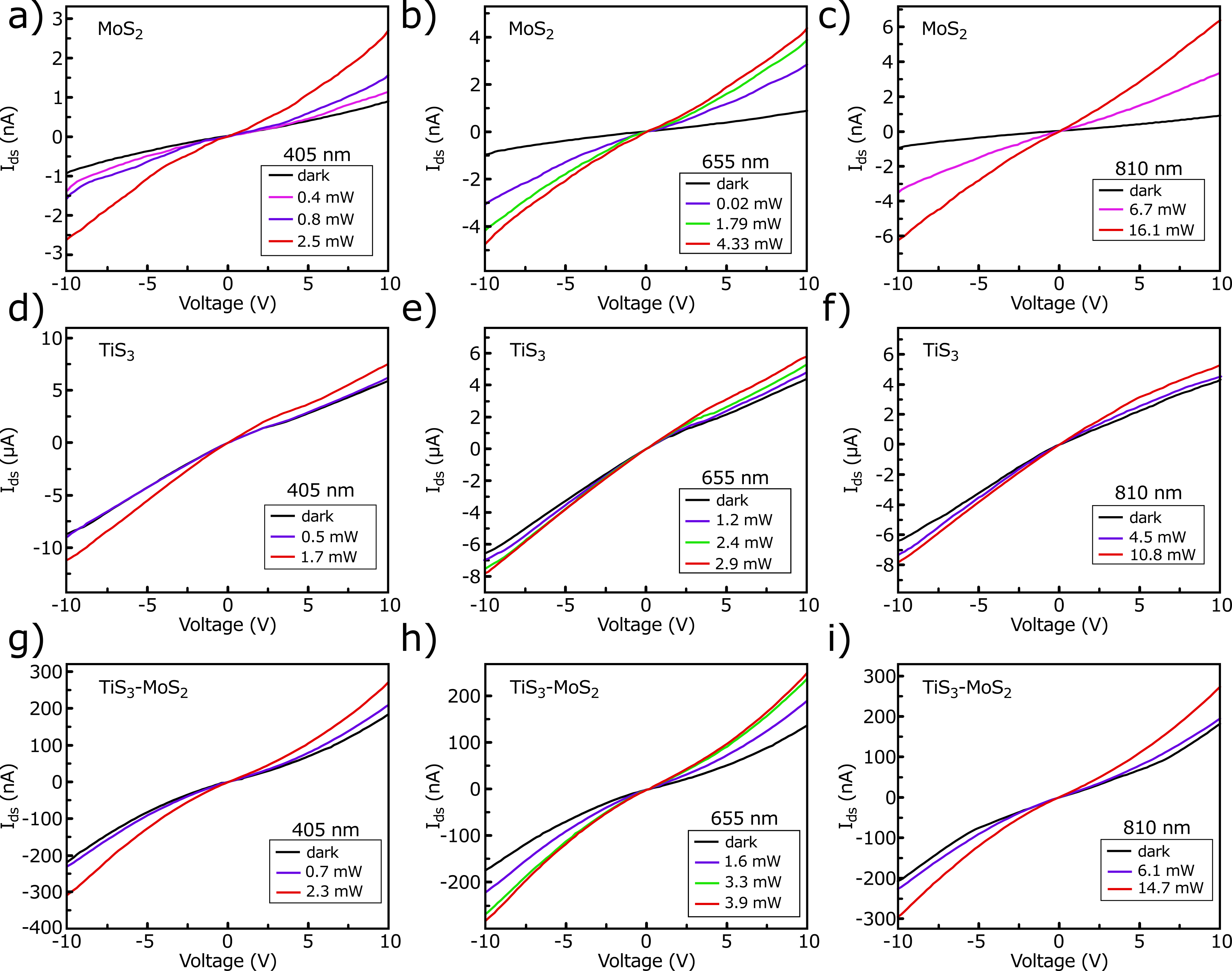


**Figure S6.** I_ds_-V characteristics of the MoS_2_, TiS_3_ and TiS_3_-MoS_2_ PDs under **a), d), and g)** 405 nm, **b), f), and h)** 655 nm, and **c), f) and i)** 810 nm laser illuminations at different power intensities, respectively.


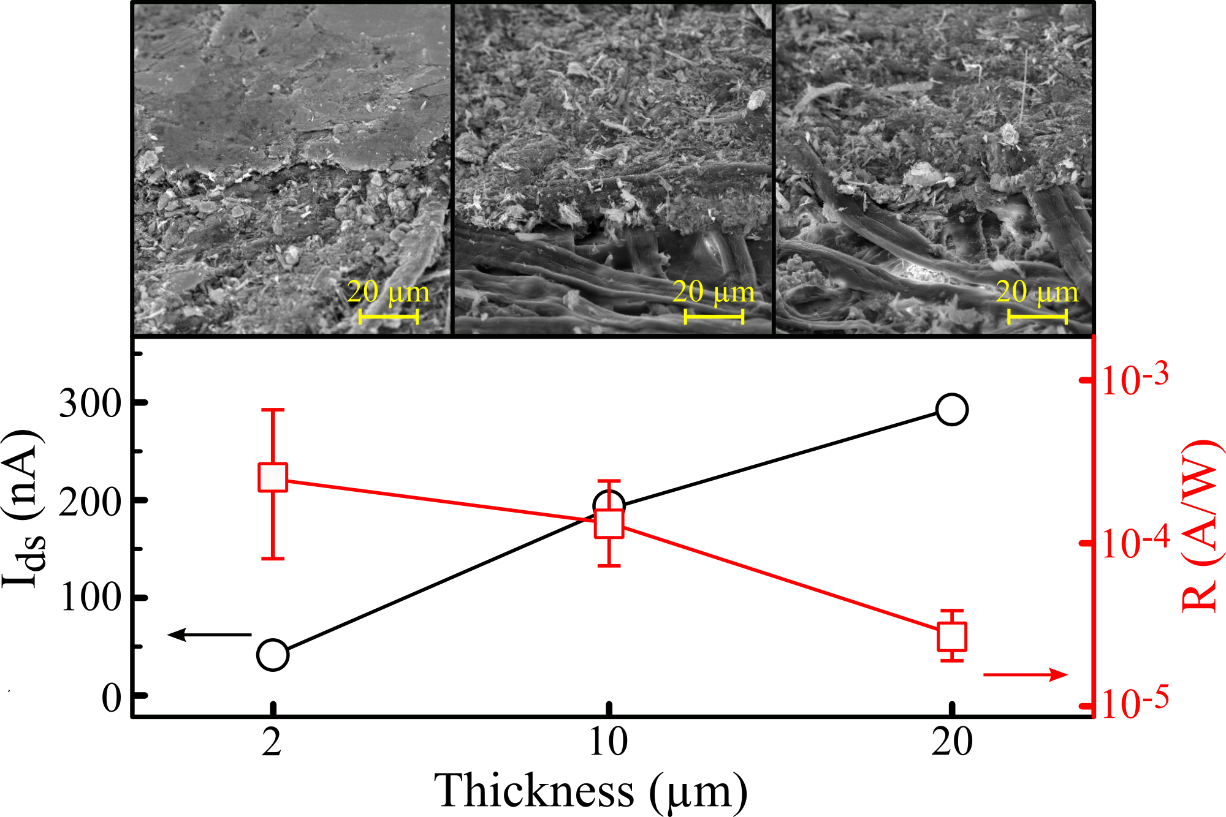


**Figure S7.** Effect of three different TiS_3_ thicknesses in dark current and photoresponsivity of the TiS_3_-MoS_2_ PDs.


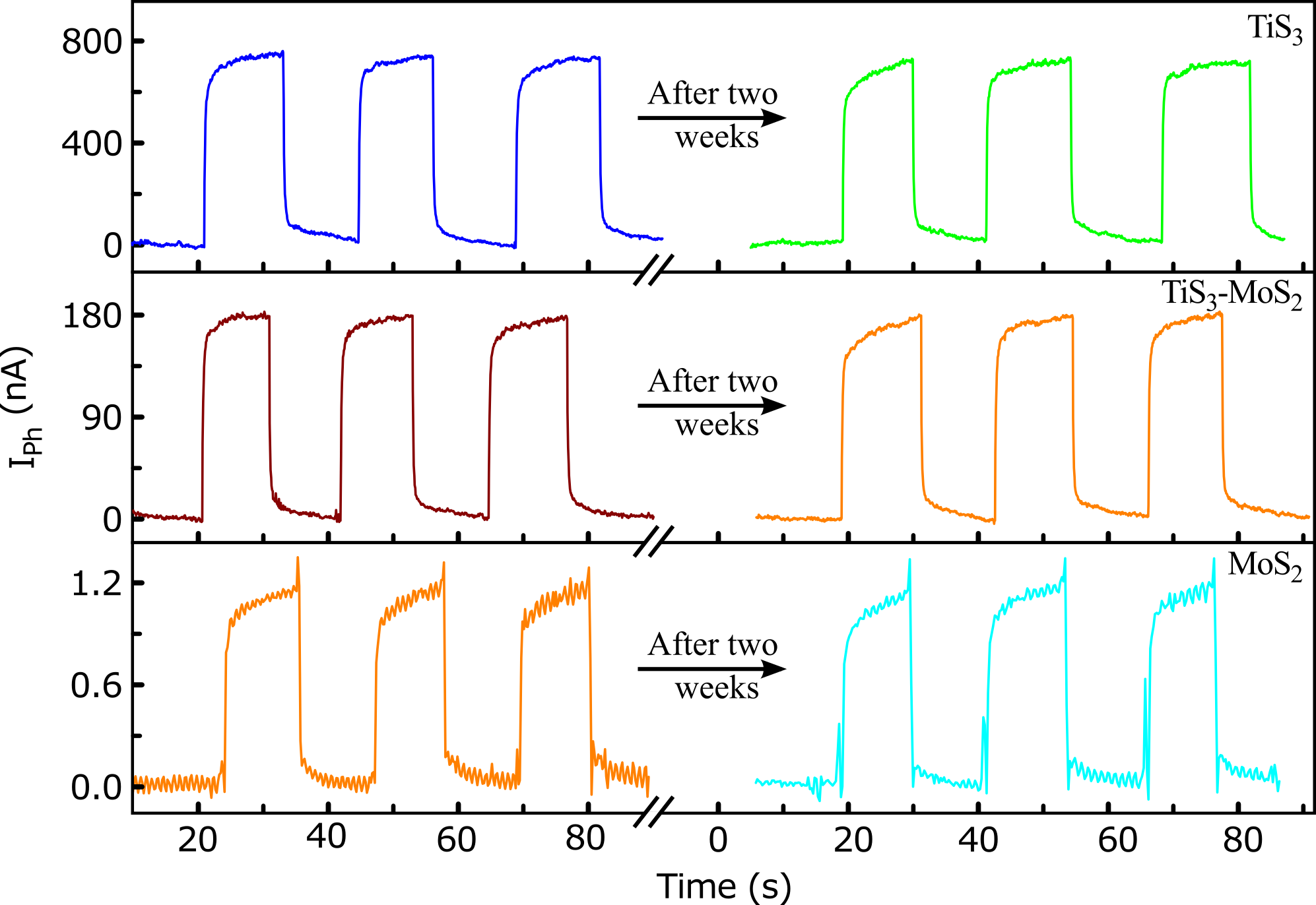


**Figure S8.** The performance of the fabricated PDs after two weeks compared with their corresponding fresh states.


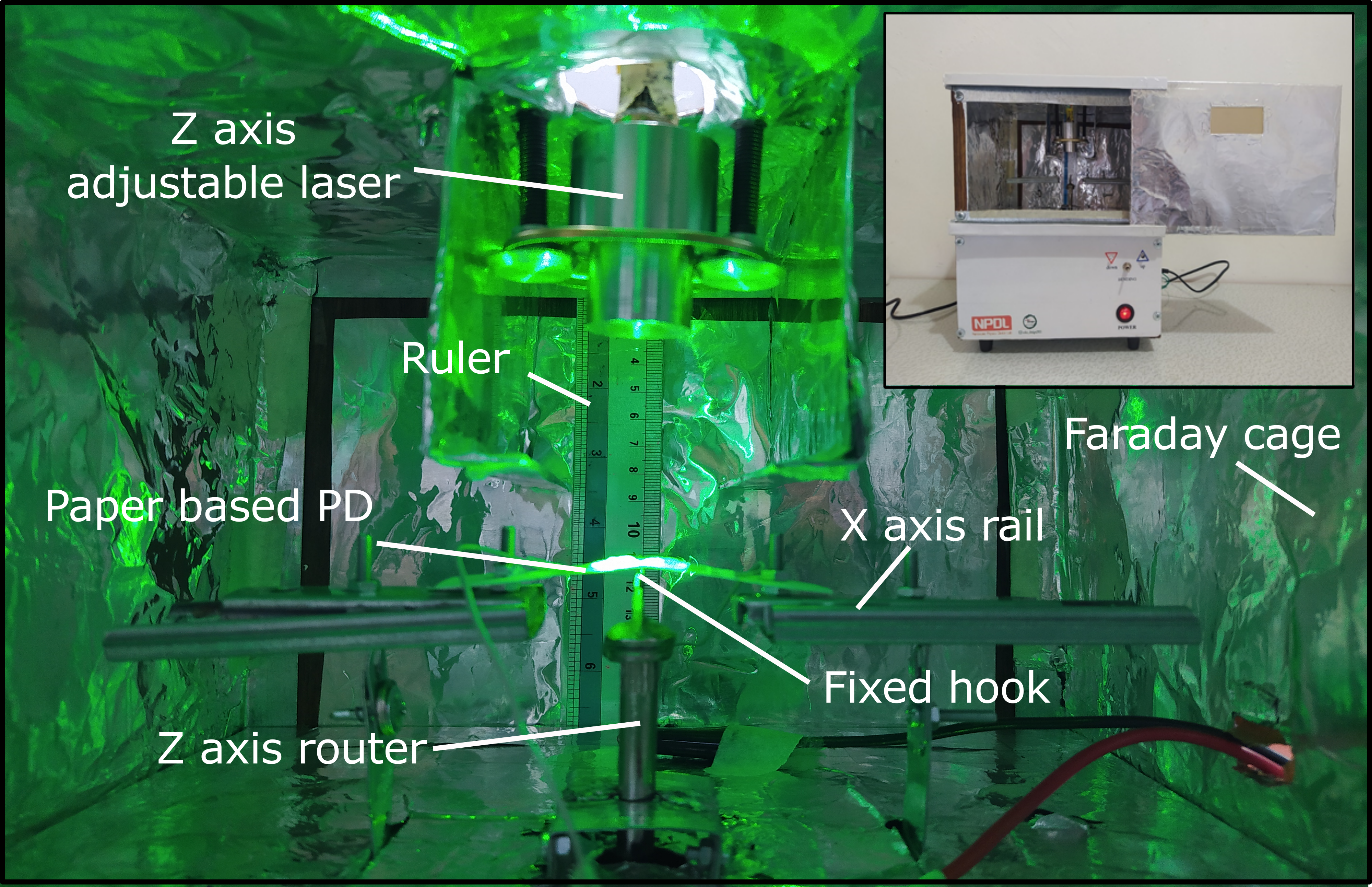


**Figure S9.** The photograph of the home-made setup to apply strain to the PDs.


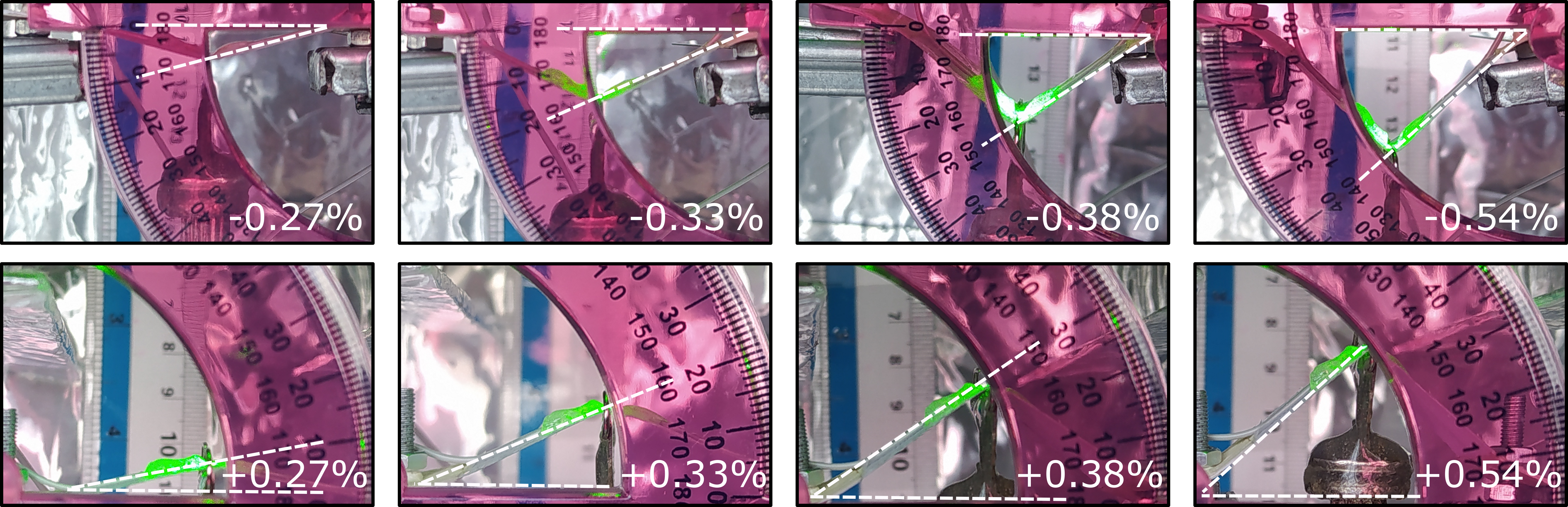


**Figure S10.** Applied strains in the range of -0.54% to +0.54% to introduced PDs.

**FEM simulation of optical absorption in direct-bandgap semiconductor:**

Finite element method is employed to address this multiphysics problem. Constitutional equations governing drift and diffusion of charge carriers in addition to physics of optical absorption in semiconductors are solved simultaneously in the devices. Wavelength of the incident light is 532 nm and the input power of the incident light varied from 0.01 W/m to 0.1 W/m, with four intensity per decade.

**Theory of optical Transition in direct-bandgap semiconductors**

Hamiltonian of an electron subject to an incident electromagnetic field can be written as[1,2]:

(S1)

$$H=\frac{1}{2m_{0}}(-i\hbar\nabla-q\mathbf{A})^{2}+V$$

Where **A** is the magnetic vector potential induced by laser beam and $m_{0}$ is the mass of the electron. The quantum mechanical Hamiltonian operator can be expressed as superposition of two terms: 1) original stationary Hamiltonian $\boldsymbol{H}_{\mathbf{0}}$ 2) a small perturbation named $\boldsymbol{H}^{\mathbf{'}}$ as follows:

(S2)

$$H=H_{0}+H^{'}$$

Therefore, we have:

$H_{0}=\frac{1}{2m_{0}}\boldsymbol{P}^{2}+V$

$H^{'}=\frac{e}{2m_{0}}\boldsymbol{A} .\boldsymbol{P}$

(S3)

Using Fermi golden rule and time-dependent perturbation theory [3,4], probability of upward transition from the ground energy state $E_{1}$ to the unoccupied energy state of $E_{2}$ can be expressed as:

$$W_{1\to2}=\frac{2\pi}{\hbar}|H_{12}^{'}|^{2} \delta(E_{2}-E_{1}-\hbar\omega_{0})$$

(S4)

Where the delta function guaranties the conservation of energy in the transitions.

Accordingly, rate of stimulated absorption which reflects the total number of upward transitions in the unit of volume per unit of time can be obtained by integration on all the possible pairs of states with energy difference equal to the energy of the incident photon, written as follows:

$$G_{stimulated}^{1\to2}=\iiint\frac{2\pi}{\hbar}|H_{12}^{avg}|^{2} g\left( k \right)f_{v}(1-f_{c}) \delta(E_{2}-E_{1}-\hbar\omega_{0})d^{3}k$$

(S5)

In the above equation, *g(k)* is the density of states in k-space and $f_{c}$ and $f_{v}$ are electron occupancy factors of conduction and valence band, respectively, given by:

$f_{c}=\frac{1}{1+exp (\frac{E_{2v}-E_{fn}}{k_{B}T})}$ $f_{v}=\frac{1}{1+exp(\frac{E_{1v}-E_{fp}}{k_{B}T})}$

(S6)

**Table S2.** Parameters used in the finite element simulation.

| Material property | Symbol [unit] | MoS_2_ | TiS_3_ |
| --- | --- | --- | --- |
| Bandgap | E_g_ [eV] | 1.6 | 1.1 |
| Electron affinity | χ_0_[V] | 4.2 | 4.7 |
| Effective density of states, valence band | N_V_ [1/cm^3] | 2.1E17 | 4.543E18 |
| Effective density of states, conduction band | N_c_ [1/cm^3] | 2.8E17 | 4.472E19 |
| Electron mobility | μ_e_  [m²/(V·s)] | 15E-4 | 10.1E-4 |
| Hole mobility | μ_h_ [m²/(V·s)] | 10E-4 | 12.1 E-4 |
| Direct recombination factor | C [cm^3/s] | 10E-10 | 5E-10 |
| Electron lifetime  (SRH model) | τ_e_ [ns] | 10 | 5 |
| Hole lifetime  (SRH model) | τ_h_ [ns] | 10 | 5 |


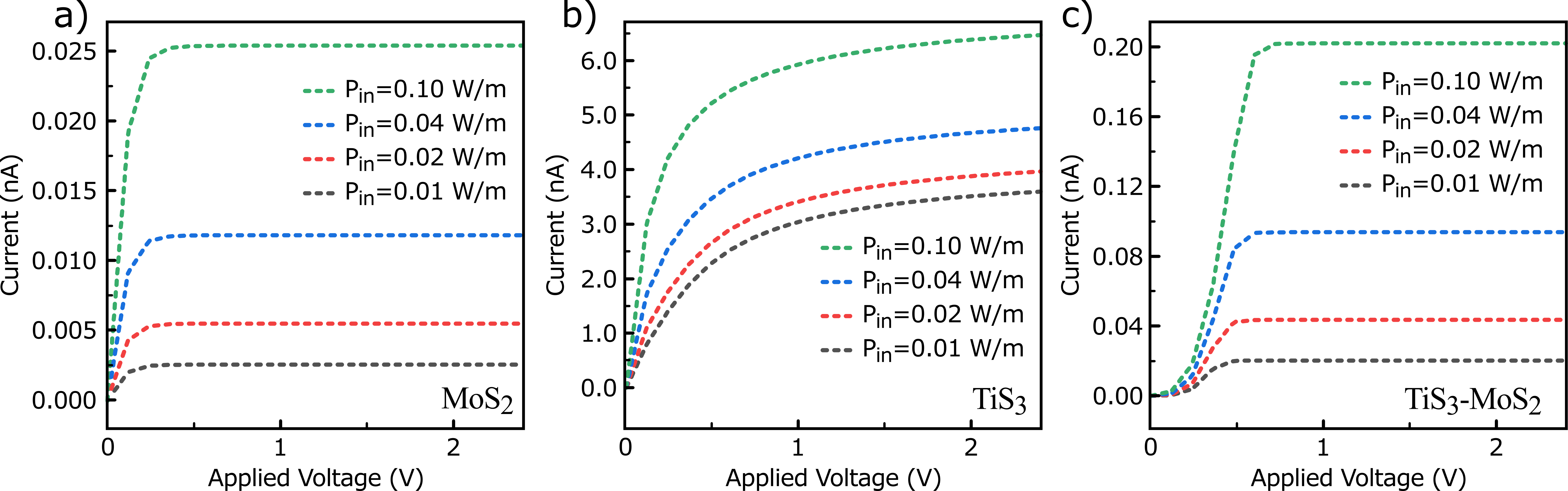


**Figure S11.** I-V characteristic curves of **a)** MoS_2_ **b)** TiS_3_ and **c)** TiS_3_-MoS_2_ PDs obtained from the simulation results. Drain-source voltage is changed from 0 to 2 V.

**References:**

[1] S.L Chuang, *Physics of Photonic Devices*, John Wiley and Sons Inc., 2009.

[2] L.D. Landau and E.M. Lifshitz, *The Classical Theory of Fields, Course of Theoretical Physics* (vol. 2), Butterworth-Heinmann, 1975.

[3] N.W. Ashcroft and D.N. Mermin, *Solid State Physics*, Harcourt Publishing, 1976.

[4] L.D. Landau and E.M. Lifshitz, *Quantum Mechanics (Non-relativistic theory), Course of Theoretical Physics* (vol. 3), Butterworth-Heinmann, 2003.
